# Integrated Analysis Identifies a TRPM4-Related Sodium Overload Signature with Prognostic Value and Experimentally Validates RNPEPL1 in Lung Adenocarcinoma

**DOI:** 10.3390/cancers18162643

**Published:** 2026-08-16

**Authors:** Wenjia Xia, Ming Li, Youtao Xu, Dongjie Feng, Wenhao Ouyang, Lin Xu

**Affiliations:** 1 Department of Thoracic Surgery, Jiangsu Cancer Hospital & Jiangsu Institute of Cancer Research & The Affiliated Cancer Hospital of Nanjing Medical University, Nanjing 210010, China; 2 Guangdong Provincial Key Laboratory of Malignant Tumor Epigenetics and Gene Regulation, Department of Medical Oncology, Sun Yat-sen Memorial Hospital, Sun Yat-sen University, Guangzhou 510030, China

**Keywords:** TRPM4, sodium overload-related cell death, lung adenocarcinoma, prognostic signature, RNPEPL1

## Abstract

**Background:** Lung adenocarcinoma (LUAD) remains a leading cause of cancer-related mortality worldwide, and effective biomarkers for prognosis and therapeutic guidance are still lacking. TRPM4, a sodium-related ion channel, has been implicated in tumor progression; however, its role in LUAD, particularly at the single-cell level, remains unclear. **Methods:** Single-cell RNA sequencing data were used to analyze the cellular distribution of TRPM4 in LUAD. TRPM4-related genes were identified through correlation analysis in the TCGA cohort, followed by construction of a prognostic model using Cox and LASSO regression analyses. The model was validated in an independent GEO dataset. Functional enrichment and drug sensitivity analyses were performed to explore the underlying mechanisms and therapeutic implications. Random forest analysis was applied to identify key genes, and in vitro experiments were conducted to validate their biological functions. **Results:** A TRPM4-related prognostic signature was established, demonstrating robust predictive performance in both training and validation cohorts. High-risk patients were characterized by activation of cell cycle and DNA replication pathways, showing differences in computationally predicted sensitivity to multiple chemotherapeutic and targeted agents. Furthermore, RNPEPL1 was identified as a key gene and experimentally validated to promote LUAD cell proliferation, migration, and invasion. **Conclusions:** The TRPM4-related signature and RNPEPL1 may provide novel insights for risk stratification and personalized therapeutic strategies.

## Simple Summary

Lung adenocarcinoma remains difficult to manage because patients with similar clinical characteristics can have markedly different outcomes and treatment responses. In this study, we investigated TRPM4, an ion channel associated with sodium overload-related cell death, using single-cell and bulk RNA sequencing data. We developed a TRPM4-related gene signature that successfully separated patients into high- and low-risk groups and predicted overall survival in both training and independent validation cohorts. High-risk tumors showed increased activity of cell cycle and DNA replication pathways and distinct predicted responses to several anticancer drugs. RNPEPL1 was identified as a key gene within the signature. Laboratory experiments showed that reducing RNPEPL1 expression inhibited lung adenocarcinoma cell growth, migration, invasion, and tumor formation, while also increasing sensitivity to selected targeted drugs. These findings may support improved prognostic assessment and help guide future personalized treatment strategies for lung adenocarcinoma.

## 1. Introduction

Lung cancer remains one of the leading causes of cancer-related mortality worldwide, and lung adenocarcinoma (LUAD) is the most common histological subtype of non-small cell lung cancer [1,2]. Despite advances in targeted therapy and immunotherapy, the prognosis of LUAD patients remains unsatisfactory, largely due to tumor heterogeneity and the lack of reliable biomarkers for prognosis and therapeutic response prediction [3,4]. Therefore, identifying novel molecular mechanisms and robust prognostic signatures is of great clinical significance.

Programmed cell death plays a critical role in tumor development and progression. Recently, sodium overload-related cell death, also referred to in some studies as necrosis by sodium overload (NECSO), has been proposed as an emerging biological process linked to ionic imbalance and cellular injury [5]. However, this concept is still relatively new, and its nomenclature and mechanistic boundaries have not yet been universally established. Therefore, in the present study, we use the term “sodium overload-related cell death” to describe this emerging biological context. TRPM4 (transient receptor potential melastatin 4), a calcium-activated non-selective cation channel, has been reported to regulate intracellular ion homeostasis and participate in various physiological and pathological processes, including cancer progression [6,7]. Emerging evidence suggests that TRPM4 may contribute to tumor proliferation, migration, and immune modulation. Nevertheless, its specific role in LUAD, especially at the single-cell level and in the context of sodium overload-related cell death, has not been systematically investigated [8,9]. With the rapid development of high-throughput sequencing technologies, single-cell RNA sequencing (scRNA-seq) provides an unprecedented opportunity to dissect tumor heterogeneity and characterize gene expression patterns at cellular resolution. In addition, integrative analysis combining bulk RNA-seq data from The Cancer Genome Atlas (TCGA) with advanced machine learning approaches enables the construction of reliable prognostic models [10].

In this study, we systematically investigated the role of TRPM4 in LUAD using single-cell and bulk RNA-seq data. A TRPM4-related prognostic model was constructed and validated, followed by functional and drug sensitivity analyses. Additionally, RNPEPL1 was identified as a key gene and experimentally confirmed to promote LUAD progression. Collectively, our findings highlight the potential of TRPM4 as a prognostic biomarker and provide insights into personalized therapeutic strategies for LUAD patients.

## 2. Materials and Methods

### 2.1. Data Collection and Preprocessing

The single-cell RNA sequencing dataset NSCLC_EMTAB6149 was obtained from the Tumor Immune Single-cell Hub 2 (TISCH2) online database (https://tisch.compbio.cn/home/ (accessed on 28 April 2026)), with the original dataset derived from ArrayExpress accession E-MTAB-6149. The transcriptome profiles and corresponding clinical information of the TCGA-LUAD cohort were downloaded from The Cancer Genome Atlas (TCGA) database and used as the training cohort. The independent GEO cohort GSE68465 was obtained from the Gene Expression Omnibus (GEO) database and used as the external validation cohort.

For the TCGA-LUAD cohort, RNA-seq expression data were normalized and log2-transformed before subsequent analyses. For the GSE68465 cohort, the processed expression matrix and corresponding clinical annotation file were downloaded from GEO. When multiple probes corresponded to the same gene symbol, their expression values were averaged. Patients without complete overall survival information, including survival time or survival status, were excluded from subsequent analyses. Gene expression profiles were further normalized when necessary to ensure comparability within each cohort. The prognostic signature was developed in the TCGA-LUAD cohort. The same risk-score formula derived from the TCGA cohort was then applied to the GSE68465 cohort to calculate the risk score for each patient.

### 2.2. Single-Cell RNA Sequencing Analysis

The NSCLC_EMTAB6149 dataset was used to evaluate the cellular distribution of TRPM4 expression in the lung cancer tumor microenvironment. According to the TISCH2 workflow, collected scRNA-seq datasets were uniformly processed using the MAESTRO pipeline, including quality control, batch effect evaluation and correction, cell clustering, differential expression analysis, cell type annotation, malignant cell classification, and gene set enrichment analysis. In the original TISCH standardized workflow, low-quality cells were removed based on library size and the number of detected genes, with cells excluded if the total UMI count was <1000 or the number of detected genes was <500. Batch effects were evaluated using an entropy-based metric and corrected when necessary. Cell clusters were annotated by TISCH2 based on canonical marker genes, original annotations, and malignant-cell identification [11]. In this study, we used the processed and annotated TISCH2 results to visualize TRPM4 expression across different cell populations.

### 2.3. Analysis of TRPM4 Expression and Identification of Related Genes

The expression distribution of TRPM4 across different cell types was visualized using UMAP plots. To identify TRPM4-related genes, Pearson correlation analysis was performed in the TCGA-LUAD dataset. Multiple testing was controlled using the Benjamini–Hochberg false discovery rate (FDR) method. Genes with an absolute correlation coefficient greater than 0.30 and FDR < 0.05 were selected as TRPM4-related genes for further analysis. This threshold was chosen to retain genes showing at least a moderate correlation with TRPM4 while reducing the inclusion of weakly associated genes with limited biological relevance.

### 2.4. Functional Enrichment Analysis

Gene Ontology (GO) and Kyoto Encyclopedia of Genes and Genomes (KEGG) enrichment analyses were conducted using the “clusterProfiler” R package. A *p* value < 0.05 was considered statistically significant. Gene set enrichment analysis (GSEA) was performed to explore the biological differences between high- and low-risk groups using the “clusterProfiler” and “enrichplot” packages.

### 2.5. Construction of the Prognostic Model

Univariate Cox regression analysis was performed to screen TRPM4-related genes associated with overall survival in the TCGA-LUAD cohort. Multiple testing was controlled using the Benjamini–Hochberg FDR method, and genes with FDR < 0.05 were considered prognostically significant [12]. LASSO Cox regression analysis was then conducted using the “glmnet” R package to further select key genes and avoid overfitting. A risk score model was constructed based on the expression levels of selected genes and their corresponding regression coefficients: Risk score = Σ (Coefficient × Gene expression). Patients were divided into high- and low-risk groups according to the median risk score.

### 2.6. Survival and Validation Analysis

Kaplan–Meier survival analysis was used to compare overall survival between high- and low-risk groups using the “survival” and “survminer” R packages. Receiver operating characteristic (ROC) curves were created, and time-dependent ROC analysis was performed using the “timeROC” R package to evaluate the predictive accuracy of the model. An independent GEO dataset was used for external validation of the prognostic model.

### 2.7. Drug Sensitivity Analysis

Drug sensitivity analysis was performed using the “pRRophetic” R package based on the Genomics of Drug Sensitivity in Cancer (GDSC) pharmacogenomic dataset. The half-maximal inhibitory concentration (IC50) values of candidate drugs were computationally estimated from transcriptomic profiles using ridge regression models implemented in pRRophetic. Patients were divided into high- and low-risk groups according to the median risk score, and differences in predicted IC50 values between the two groups were compared using the Wilcoxon rank-sum test. Predicted IC50 distributions were summarized using boxplots showing median values and interquartile ranges to indicate the magnitude of the predicted differences where applicable. Because this analysis was based on cell-line pharmacogenomic models rather than clinical treatment-response data from LUAD patients, the results were considered exploratory and hypothesis-generating.

### 2.8. Molecular Docking Analysis

Molecular docking was performed to evaluate the potential binding between RNPEPL1 and selected targeted agents. The three-dimensional structure of RNPEPL1 was obtained from the AlphaFold Protein Structure Database using UniProt ID Q9HAU8. The ligand structures of gefitinib (PubChem CID: 123631), erlotinib (PubChem CID: 176870), and crizotinib (PubChem CID: 11626560) were downloaded from the PubChem database. Ligand structures were prepared and energy-minimized using ChemDraw 20.0. The protein structure was preprocessed using PyMOL 2.4 and AutoDockTools V4.2.6, including removal of redundant molecules, addition of hydrogen atoms, and conversion to PDBQT format. Molecular docking was conducted using AutoDock Vina 1.1.2 with an exhaustiveness value of 8. The docking grid was centered at x = −3.3661, y = −9.3802, and z = −3.5659, with grid box dimensions of 78.9675 × 99.5236 × 82.3701 Å. The docking pose with the lowest binding energy was selected for further visualization. Protein–ligand interactions were analyzed using PyMOL and Discovery Studio 2019.

### 2.9. Random Forest Analysis

A random forest model was constructed using the “randomForest” R package to evaluate the importance of prognostic genes. The number of trees was optimized based on the model error rate. Genes were ranked according to their importance scores, and the top-ranked gene was selected for further experimental validation.

### 2.10. In Vitro Drug Sensitivity and IC_50_ Assay

To validate the predicted drug sensitivity results, in vitro drug sensitivity assays were performed in LUAD cells. PC9 cells were used to evaluate the sensitivity to gefitinib and erlotinib, while H3122 cells were used to evaluate the sensitivity to crizotinib. Briefly, cells transfected with siRNPEPL1-1, siRNPEPL1-2, or siNC were seeded into 96-well plates at an appropriate density and allowed to adhere overnight. After cell attachment, PC9 cells were treated with increasing concentrations of gefitinib or erlotinib, and H3122 cells were treated with increasing concentrations of crizotinib for 48 h.

Cell viability was assessed using the Cell Counting Kit-8 (CCK-8), Kumamoto, Japan) according to the manufacturer’s instructions. Briefly, 10 μL of CCK-8 solution was added to each well and incubated for 1–2 h at 37 °C. The absorbance was measured at 450 nm using a microplate reader. Cell viability was calculated relative to the untreated control group. Dose–response curves were generated based on the relative cell viability at different drug concentrations, and the half-maximal inhibitory concentration (IC_50_) values were calculated using nonlinear regression analysis. All experiments were performed in triplicate.

### 2.11. Cell Culture

Lung adenocarcinoma cell lines A549, PC9, and H3122 were obtained from the Cell Bank of the Chinese Academy of Sciences (Shanghai, China). Cells were cultured in RPMI-1640 medium (Waltham, MA, USA) supplemented with 10% fetal bovine serum (FBS) (Waltham, MA, USA) and 1% penicillin–streptomycin. Cells were maintained at 37 °C in a humidified incubator with 5% CO_2_. The culture medium was replaced every 2–3 days, and cells were passaged when they reached approximately 80–90% confluence.

### 2.12. siRNA Transfection

Small interfering RNAs (siRNAs) targeting RNPEPL1, including siRNPEPL1-1 and siRNPEPL1-2, and a negative control siRNA (siNC) were synthesized by GenePharma (Shanghai, China). The siRNA sequences were as follows: siRNPEPL1-1, sense 5′-GGUGGACCCUUACACGATT-3′ and antisense 5′-UCGUGAAGGUGCCACCTT-3′; siRNPEPL1-2, sense 5′-CGCCAUGAUUUCACCUATT-3′ and antisense 5′-UAGGUGAAAUGAUGGCGTT-3′; siNC, sense 5′-UUCUCCGAACGUGUCACGUTT-3′ and antisense 5′-ACGUGACACGUUCGGAGAATT-3′. Cells were seeded into 6-well plates and transfected at approximately 60–70% confluence using Lipofectamine 3000 (Waltham, MA, USA) according to the manufacturer’s instructions. Briefly, siRNA and transfection reagent were diluted separately in Opti-MEM medium and then mixed gently to form siRNA–lipid complexes, which were added to the cells. After 4–6 h, the medium was replaced with fresh complete medium. Cells were harvested 24–48 h post-transfection for subsequent experiments.

### 2.13. Quantitative Real-Time PCR (qRT–PCR)

Total RNA was extracted from cells using TRIzol reagent (Waltham, MA, USA) according to the manufacturer’s protocol. RNA concentration and purity were assessed using a NanoDrop spectrophotometer. Complementary DNA (cDNA) was synthesized using a reverse transcription kit, such as PrimeScript RT reagent kit (Kusatsu, Japan). Quantitative real-time PCR was performed using SYBR Green Master Mix (Kusatsu, Japan) on an Applied Biosystems 7500 real-time PCR system. The primer sequences were as follows: RNPEPL1, forward 5′-CAGTCATCCTGGGTACAC-3′ and reverse 5′-GTGTGCAAGCACGGGAAGAA-3′; β-actin, forward 5′-CACCACCTCCTCCACCTTGA-3′ and reverse 5′-TCTCTCTTCCTCTTGGCTCTTGG-3′. The amplification conditions were as follows: initial denaturation at 95 °C for 30 s, followed by 40 cycles of 95 °C for 5 s and 60 °C for 30 s. Relative gene expression levels were normalized to β-actin and calculated using the 2^−ΔΔCt^ method. All experiments were performed in triplicate.

### 2.14. Colony Formation Assay

Transfected cells were seeded into 6-well plates at a density of approximately 500–1000 cells per well and cultured for 10–14 days. The culture medium was replaced every 3 days. After colony formation, cells were fixed with 4% paraformaldehyde for 15 min and stained with 0.1% crystal violet for 15–20 min. Colonies containing more than 50 cells were counted under a microscope. Each experiment was performed at least three times independently.

### 2.15. Transwell Migration and Invasion Assays

Cell migration and invasion assays were performed using Transwell chambers (8 μm pore size, New York, NY, USA). For the migration assay, transfected cells were suspended in serum-free medium, and approximately 5 × 10^4^ cells were seeded into the upper chamber. Medium containing 10% FBS was added to the lower chamber as a chemoattractant. For the invasion assay, the upper chamber was pre-coated with Matrigel (Franklin Lakes, NJ, USA) before seeding the cells. After incubation for 24–48 h, cells on the upper surface of the membrane were removed, while cells that migrated or invaded to the lower surface were fixed with 4% paraformaldehyde and stained with 0.1% crystal violet. The stained cells were counted in five randomly selected fields under a light microscope.

### 2.16. In Vivo Xenograft Assay

To further evaluate the biological role of RNPEPL1 in LUAD tumor growth in vivo, a xenograft mouse model was established. LUAD cells with stable RNPEPL1 knockdown (shRNPEPL1) and corresponding control cells (shControl) were generated using lentiviral short hairpin RNA transduction. Female BALB/c nude mice were randomly divided into the shControl and shRNPEPL1 groups. Cells were suspended in PBS and subcutaneously injected into the flank of each mouse. Tumor growth was monitored every two days, and tumor volume was calculated using the formula: volume = length × width^2^/2. At the end of the experiment, mice were sacrificed, and tumors were excised, photographed, and weighed. All animal experiments were performed in accordance with institutional guidelines and approved by the Ethics Committee of Jiangsu Cancer Hospital.

### 2.17. Statistical Analysis

All statistical analyses were performed using R software v4.5.2 and GraphPad Prism v10.2.0. Differences between groups were analyzed using Student’s *t*-test, one-way ANOVA, or the Wilcoxon rank-sum test, as appropriate. Data from in vitro experiments are presented as the mean ± standard deviation (SD) from at least three independent experiments. A two-sided *p* value < 0.05 was considered statistically significant.

## 3. Results

### 3.1. Cellular Distribution of TRPM4 and Identification of TRPM4-Related Genes in LUAD

**Figure 1:**
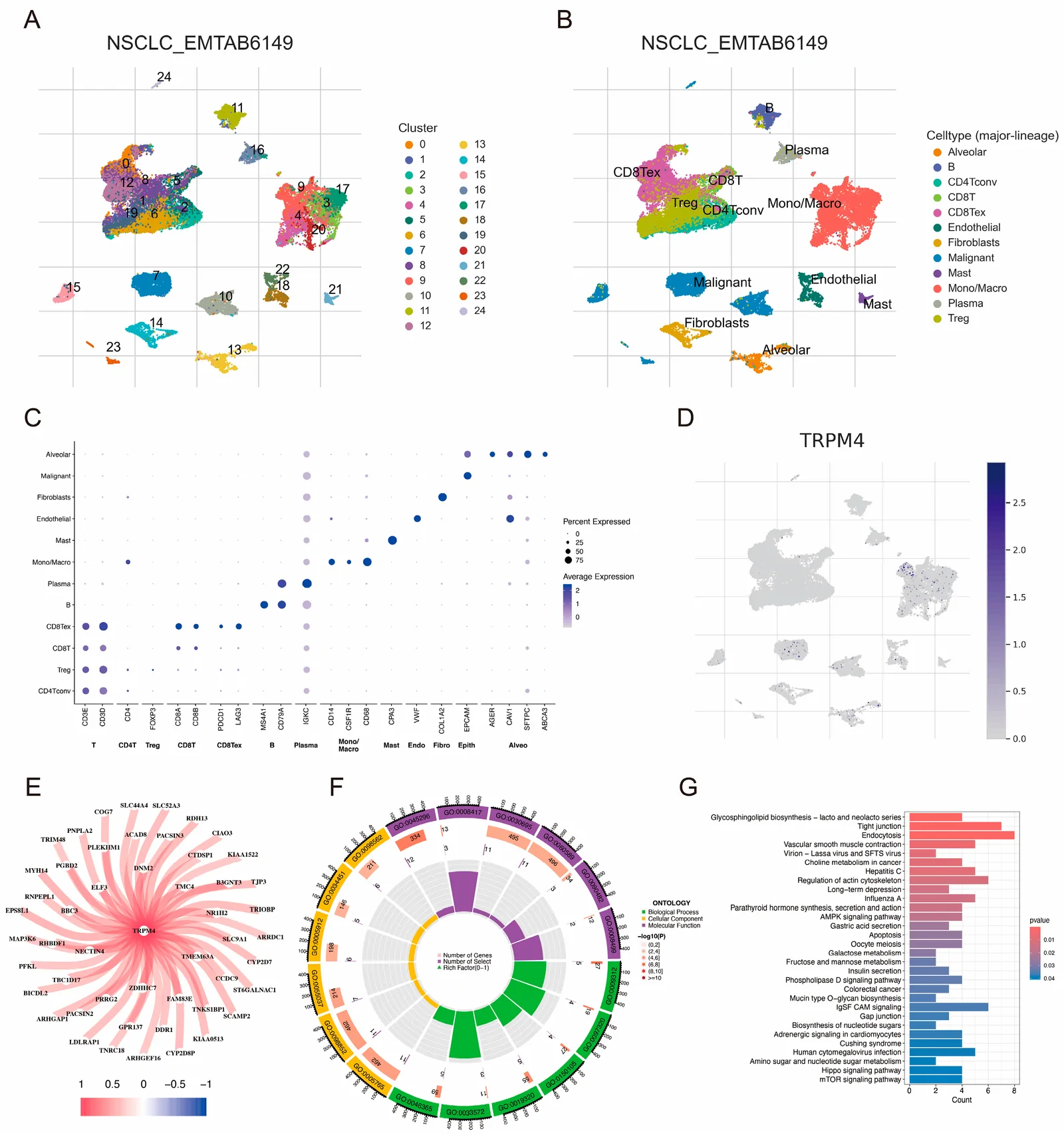
Cellular distribution and functional analysis of TRPM4 in LUAD. (**A**) UMAP visualization of single-cell RNA-seq data from the EMTAB6149 dataset showing 25 distinct cell clusters. (**B**) Annotation of cell clusters into major cell types based on canonical marker genes. (**C**) Dot plot showing the expression of representative marker genes across different cell types. (**D**) UMAP plot illustrating the expression distribution of TRPM4 across all cell populations. (**E**) Network plot of genes significantly correlated with TRPM4 in the TCGA-LUAD cohort. (**F**) Gene Ontology (GO) enrichment analysis of TRPM4-related genes. (**G**) KEGG pathway enrichment analysis of TRPM4-related genes.

To investigate the cellular distribution of TRPM4—a sodium overload-related cell death-associated gene—in the tumor microenvironment, processed and annotated single-cell RNA-sequencing data from the NSCLC_EMTAB6149 dataset in TISCH2 were analyzed. Multiple distinct cell populations were visualized and annotated into major cell types by TISCH2, including malignant, immune, and stromal cells (Figure 1A–C).

Subsequently, the expression pattern of TRPM4 across different cell populations was examined. As shown in Figure 1D, TRPM4 exhibited cell type-specific expression, with relatively higher expression levels observed in malignant cells and certain immune cell subsets, suggesting a potential role in tumor progression and immune regulation. To further explore the biological significance of TRPM4 in lung adenocarcinoma (LUAD), a correlation analysis was performed using the TCGA-LUAD cohort. A set of genes significantly correlated with TRPM4 expression was identified and visualized as a gene interaction network (Figure 1E), indicating that TRPM4 may be involved in complex regulatory networks.

Functional enrichment analyses were then conducted based on these TRPM4-related genes. The Gene Ontology (GO) analysis revealed significant enrichment in biological processes related to signal transduction, cellular response, and metabolic regulation, along with enrichment in membrane-associated components and binding activities (Figure 1F). Furthermore, KEGG pathway analysis demonstrated that these genes were mainly enriched in tumor-related and metabolism-associated pathways, including the mTOR signaling pathway, the Hippo signaling pathway, the calcium signaling pathway, and glycosphingolipid biosynthesis (Figure 1G). These findings suggest that TRPM4 may contribute to LUAD progression through multiple biological processes and signaling pathways.

### 3.2. Identification of Prognostic TRPM4-Related Genes in LUAD

**Figure 2:**
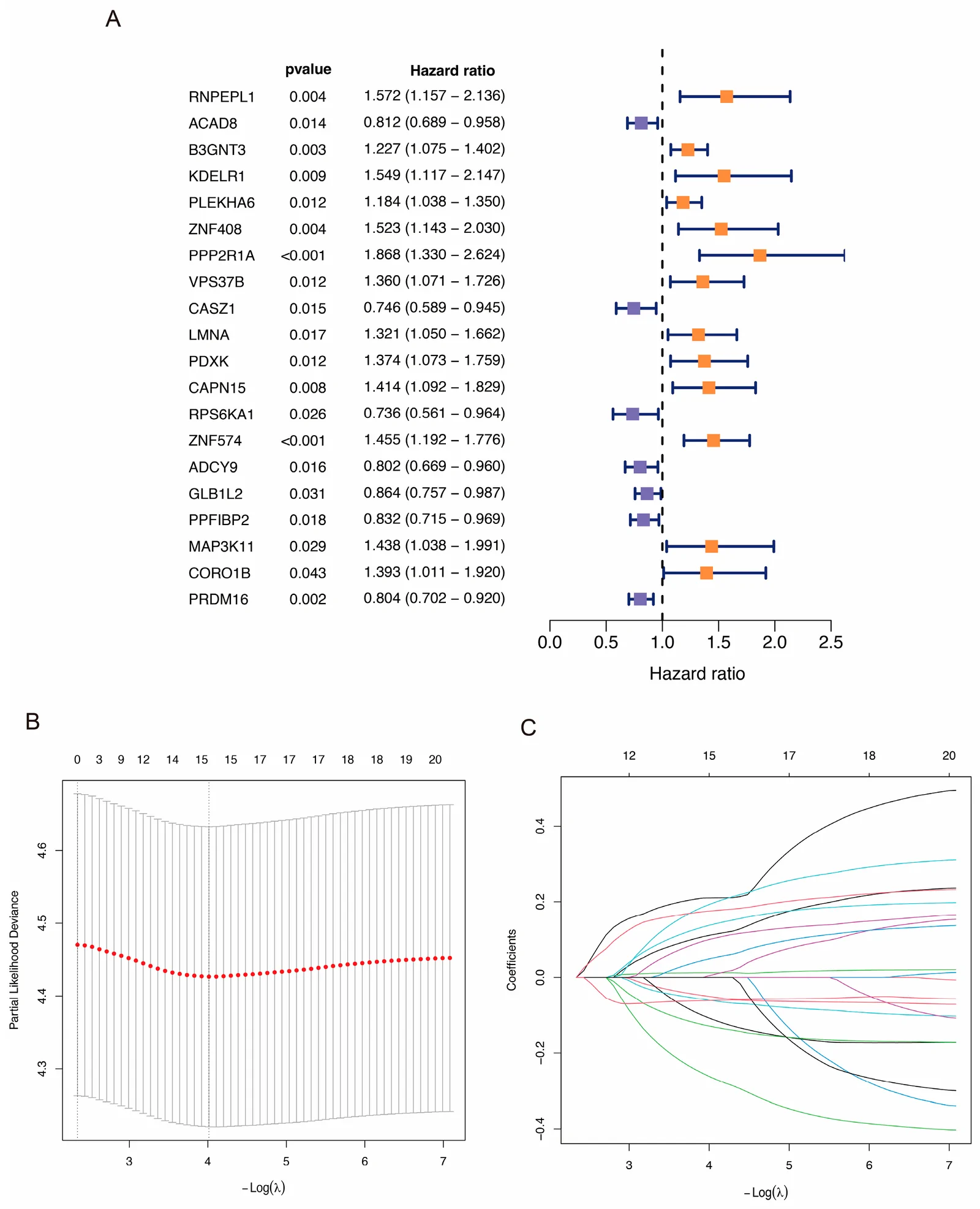
Screening of prognostic TRPM4-related genes in LUAD. Screening of prognostic TRPM4-related genes in LUAD. (**A**) Forest plot of univariate Cox regression analysis showing TRPM4-related genes significantly associated with overall survival in LUAD. (**B**) Ten-fold cross-validation for tuning parameter selection in the LASSO Cox regression model. (**C**) LASSO coefficient profiles of prognostic TRPM4-related genes in LUAD.

To identify TRPM4-related genes associated with the prognosis of lung adenocarcinoma (LUAD), univariate Cox regression analysis was first performed in the TCGA-LUAD cohort. As shown in Figure 2A, multiple TRPM4-related genes were significantly associated with overall survival. Among them, several genes exhibited hazard ratios (HRs) greater than 1, indicating potential risk factors, while others showed HRs less than 1, suggesting protective roles in LUAD progression. To further refine the prognostic gene set and avoid overfitting, least absolute shrinkage and selection operator (LASSO) Cox regression analysis was conducted. Ten-fold cross-validation was used to determine the optimal penalty parameter λ, and the minimum criterion, corresponding to lambda.min, was applied for model construction (Figure 2B). The optimal λ value was 0.015, and 15 prognostic genes with non-zero coefficients were retained, including RNPEPL1, ACAD8, B3GNT3, PLEKHA6, ZNF408, PPP2R1A, CASZ1, LMNA, PDXK, CAPN15, RPS6KA1, ZNF574, ADCY9, PPFIBP2, and PRDM16 (Figure 2C). These genes were subsequently used for risk-score calculation and prognostic model construction.

### 3.3. Construction and Validation of a TRPM4-Related Prognostic Signature in LUAD

**Figure 3:**
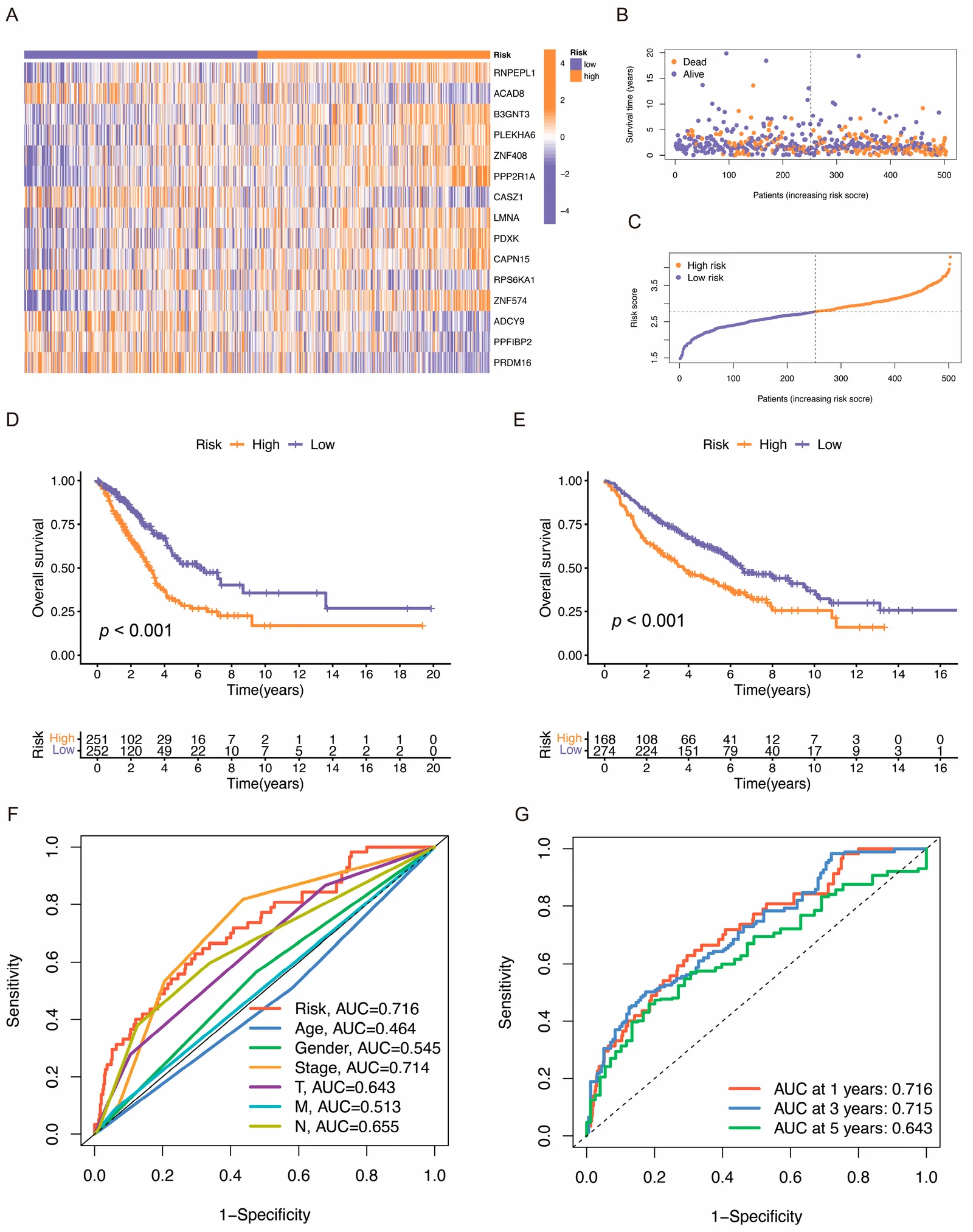
Construction and validation of the TRPM4-related prognostic model in LUAD. (**A**) Heatmap showing the expression of prognostic genes in high- and low-risk groups. (**B**) Distribution of survival status of LUAD patients ranked by risk score. (**C**) Risk score distribution of patients in the cohort. (**D**) Kaplan–Meier survival analysis of high- and low-risk groups in the TCGA training cohort. (**E**) Kaplan–Meier survival analysis of high- and low-risk groups in the GEO validation cohort. (**F**) ROC curves comparing the predictive performance of the risk score and clinical variables. (**G**) Time-dependent ROC curves at 1, 3, and 5 years.

Based on the prognostic TRPM4-related genes identified by LASSO Cox regression, a risk model was constructed to predict the overall survival of LUAD patients. The expression patterns of these genes across patients, along with their corresponding risk groups, are shown in Figure 3A. Distinct expression differences were observed between the high-risk and low-risk groups.

Patients were then stratified into high- and low-risk groups according to the median risk score. The distribution of survival status indicated that patients with higher risk scores exhibited a higher mortality rate and shorter survival time (Figure 3B). Additionally, the risk score distribution plot demonstrated a clear separation between the two groups (Figure 3C). Kaplan–Meier survival analysis revealed that patients in the high-risk group had significantly worse overall survival compared to those in the low-risk group in the TCGA training cohort (Figure 3D, *p* < 0.001). To further validate the robustness of the model, an external GEO dataset was used as the validation cohort, and similar results were observed, with the high-risk group showing significantly poorer survival outcomes (Figure 3E, *p* < 0.001). The baseline clinicopathological characteristics of the TCGA training cohort and GSE68465 validation cohort are summarized in Table S1.

To evaluate the predictive performance of the model, receiver operating characteristic (ROC) curve analysis was performed. The risk score demonstrated superior predictive ability compared with traditional clinical features such as age, gender, and TNM stage (Figure 3F). Furthermore, time-dependent ROC analysis showed that the model achieved satisfactory predictive accuracy, with AUC values of 0.716, 0.715, and 0.643 at 1, 3, and 5 years, respectively (Figure 3G). These results indicate that the TRPM4-related prognostic signature has reliable predictive value for LUAD survival. To further evaluate whether the TRPM4-related risk score was an independent prognostic factor, univariate and multivariate Cox regression analyses were performed in the TCGA cohort. In the univariate Cox analysis, the risk score was significantly associated with overall survival (HR = 3.796, 95% CI = 2.548–5.654, *p* < 0.001), together with overall stage and T, M, and N stages (Figure S1A). In the multivariate Cox analysis adjusted for age, gender, overall stage, and T, M, and N stages, the risk score remained independently associated with overall survival (HR = 3.472, 95% CI = 2.290–5.265, *p* < 0.001; Figure S1B). These results suggest that the TRPM4-related risk score may provide prognostic information independent of conventional clinicopathological factors.

### 3.4. Functional Enrichment Analysis of the High-Risk Group

To further explore the potential biological mechanisms underlying the prognostic differences between high- and low-risk groups, gene set enrichment analysis (GSEA) was performed.

**Figure 4:**
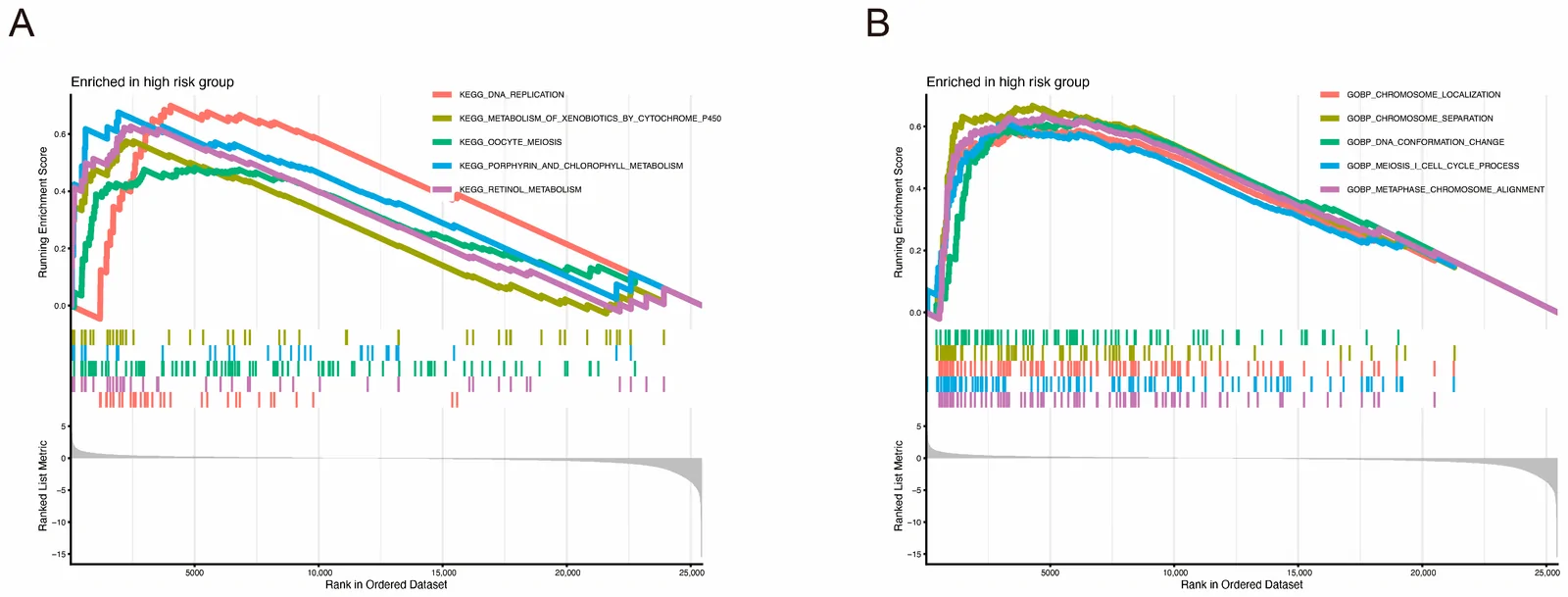
GSEA of biological pathways in the high-risk group. (**A**) KEGG pathway enrichment analysis showing significantly enriched pathways in the high-risk group. (**B**) GO enrichment analysis showing biological processes enriched in the high-risk group.

KEGG pathway analysis revealed that multiple pathways were significantly enriched in the high-risk group, including DNA replication, metabolism of xenobiotics by cytochrome P450, oocyte meiosis, porphyrin and chlorophyll metabolism, and retinol metabolism (Figure 4A). These results suggest that high-risk patients exhibit enhanced proliferative activity and metabolic reprogramming.

Consistently, Gene Ontology (GO) enrichment analysis demonstrated that genes in the high-risk group were mainly involved in cell cycle-related biological processes, such as chromosome localization, chromosome separation, DNA conformation change, meiotic cell cycle processes, and metaphase chromosome alignment (Figure 4B). Taken together, these findings indicate that the high-risk group is characterized by activation of cell cycle progression and DNA replication-related pathways, which may contribute to tumor aggressiveness and poor clinical outcomes.

### 3.5. Potential Drug Sensitivity in High- and Low-Risk Patients

**Figure 5:**
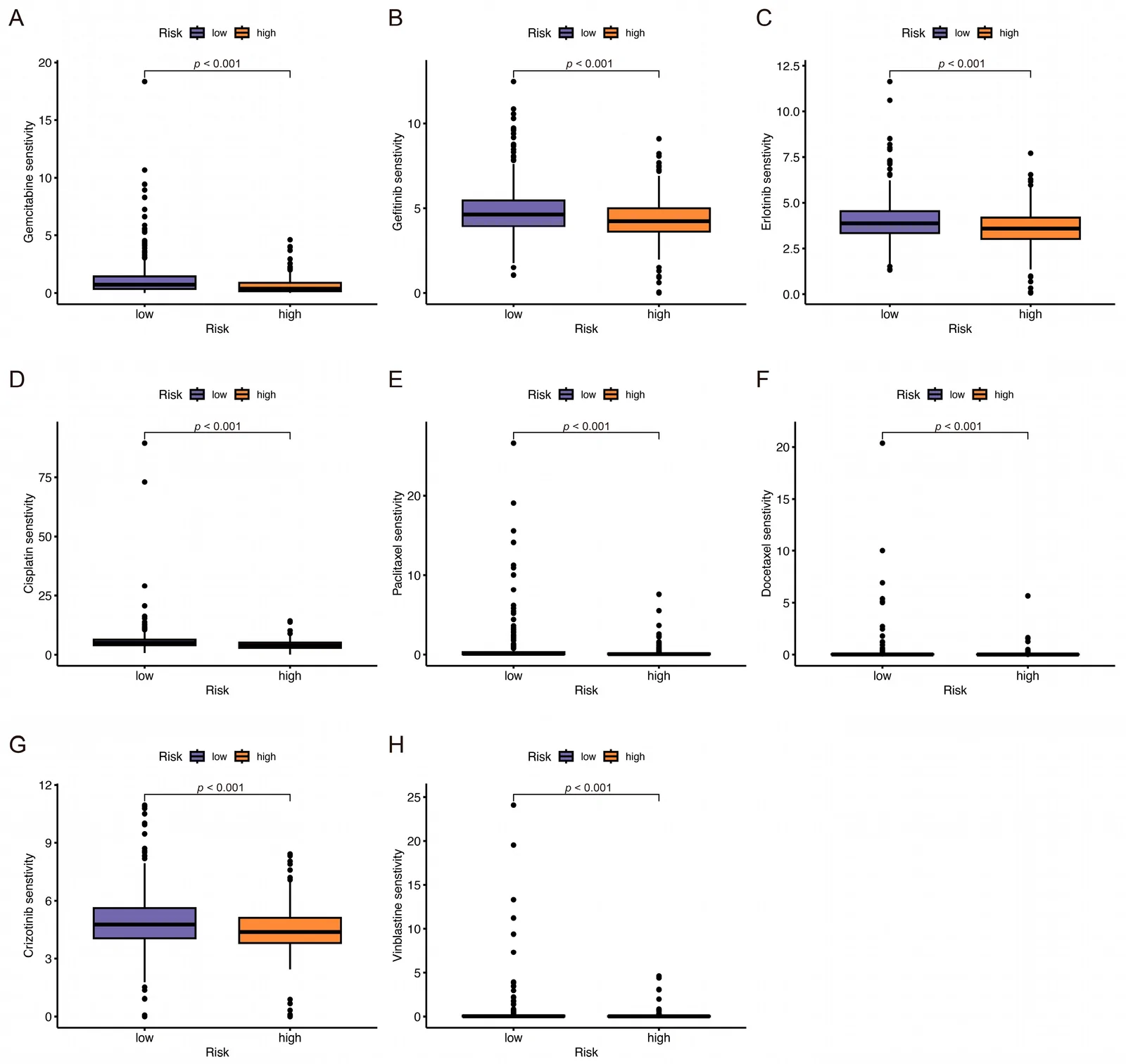
Predicted drug sensitivity analysis between high- and low-risk groups. (**A**–**H**) Comparison of computationally predicted IC50 values for FDA-approved drugs for lung cancer between the high- and low-risk groups, including gemcitabine (**A**), gefitinib (**B**), erlotinib (**C**), cisplatin (**D**), paclitaxel (**E**), docetaxel (**F**), crizotinib (**G**), and vinblastine (**H**).

To further explore the clinical significance of the TRPM4-related prognostic signature, we compared the computationally predicted IC50 values of FDA-approved drugs for lung cancer between the high- and low-risk groups using the pRRophetic package. As shown in Figure 5, the predicted IC50 distributions differed between the two risk groups for multiple therapeutic agents. Specifically, the low-risk group showed lower predicted IC50 values for gemcitabine, gefitinib, erlotinib, cisplatin, paclitaxel, docetaxel, crizotinib, and vinblastine compared with the high-risk group (Figure 5A–H). These computational predictions suggest that the TRPM4-related risk model may be associated with differential drug-response patterns at the transcriptomic level. However, because these results were inferred from GDSC-based cell-line pharmacogenomic models rather than observed clinical treatment responses, they should be interpreted as exploratory and hypothesis-generating. Further validation in clinically annotated cohorts with treatment-response data is required.

### 3.6. Identification and Experimental Validation of a Key Prognostic Gene Using Random Forest Analysis

**Figure 6:**
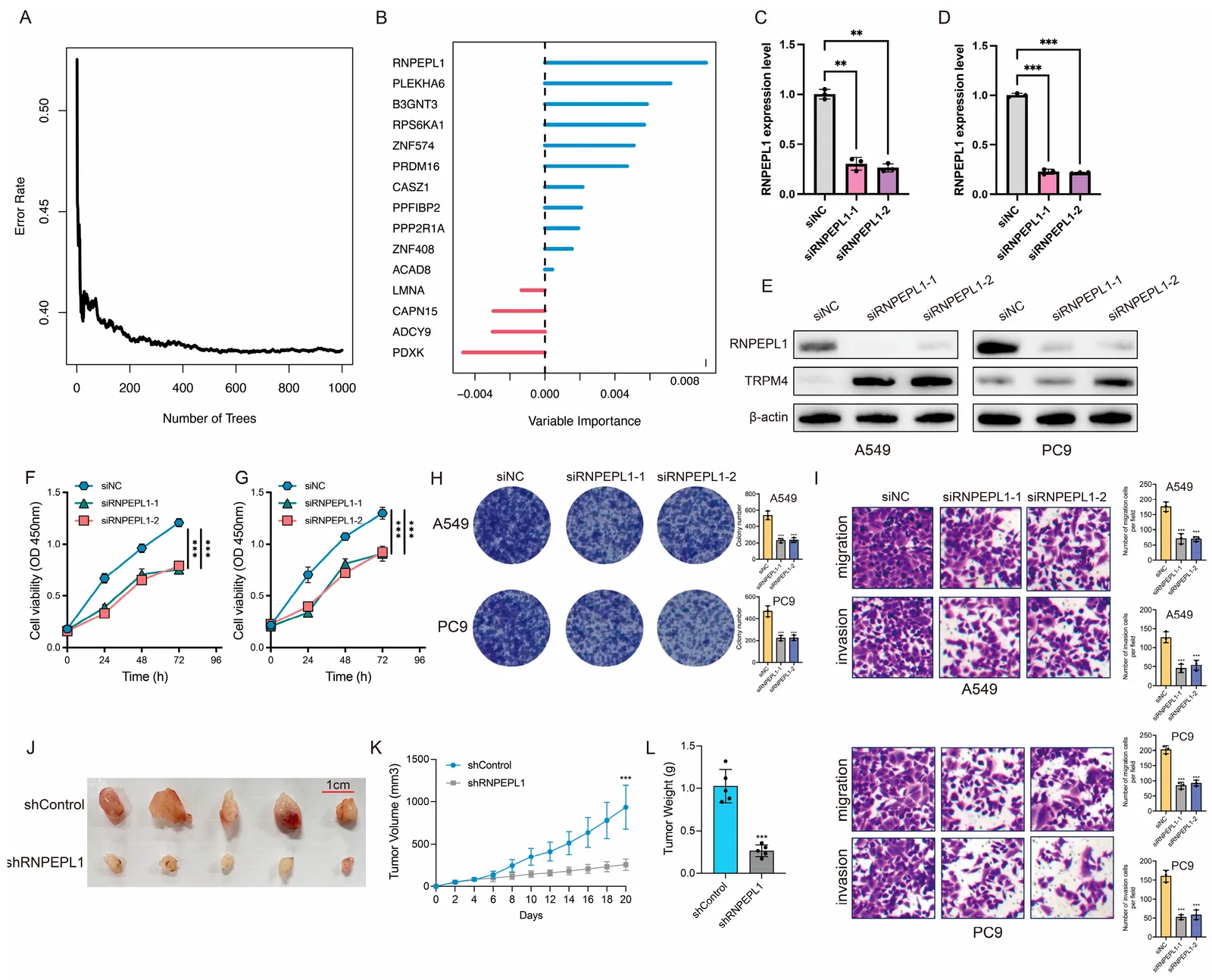
Random forest analysis and functional validation of RNPEPL1 in LUAD cells. (**A**) Error rate plot of the random forest model as the number of trees increased. (**B**) Variable importance plot showing the contribution of prognostic genes to the model. (**C**,**D**) qRT-PCR validation of RNPEPL1 knockdown efficiency in A549 and PC9 cells after siRNA transfection. (**E**) Western blot analysis confirming the knockdown efficiency of RNPEPL1 at the protein level in LUAD cells. (**F**,**G**) CCK-8 assays showing the effect of RNPEPL1 silencing on the viability of A549 and PC9 cells. (**H**) Colony formation assays showing the effect of RNPEPL1 knockdown on the clonogenic ability of A549 and PC9 cells. (**I**) Transwell assays evaluating the effects of RNPEPL1 silencing on the migration and invasion abilities of A549 and PC9 cells. (**J**) Representative images of xenograft tumors from the shControl and shRNPEPL1 groups. (**K**) Tumor growth curves showing reduced tumor volume in the shRNPEPL1 group. (**L**) Tumor weight analysis showing significantly decreased tumor weight after RNPEPL1 knockdown. Data are presented as the mean ± SD. ** *p* < 0.01, *** *p* < 0.001.

To further identify the key genes contributing to the TRPM4-related prognostic model, a random forest algorithm was applied to evaluate the importance of each model gene. As shown in Figure 6A, the model error gradually decreased and stabilized with the increasing number of trees, indicating good model performance and stability. Variable importance analysis demonstrated that RNPEPL1 had the highest contribution to the prognostic model among all candidate genes (Figure 6B). Specifically, RNPEPL1 showed the highest relative importance score (0.998), followed by PLEKHA6 (0.779), B3GNT3 (0.634), RPS6KA1 (0.616), ZNF574 (0.552), and PRDM16 (0.510). Based on its top-ranked contribution to the prognostic model, RNPEPL1 was selected for further experimental validation. Therefore, RNPEPL1 was selected for further experimental validation.

To investigate the biological role of RNPEPL1 in LUAD, siRNA-mediated knockdown was performed in A549 and PC9 cells. The knockdown efficiency of RNPEPL1 was first confirmed by qRT-PCR, showing that both siRNAs significantly reduced RNPEPL1 mRNA expression compared with the negative control group in A549 and PC9 cells (Figure 6C,D). Consistently, Western blot analysis further confirmed that RNPEPL1 protein expression was markedly decreased after siRNA transfection (Figure 6E, Figures S2 and S3).

Next, CCK-8 assays were conducted to assess cell proliferation after RNPEPL1 silencing. The results showed that knockdown of RNPEPL1 significantly inhibited the viability of both A549 and PC9 cells compared with the control group (Figure 6F,G). Consistently, colony formation assays demonstrated that RNPEPL1 silencing markedly reduced the clonogenic capacity of LUAD cells (Figure 6H). In addition, Transwell assays revealed that the migration and invasion abilities of A549 and PC9 cells were significantly suppressed after RNPEPL1 knockdown (Figure 6I). To further validate the biological significance of RNPEPL1 in LUAD, we established a subcutaneous xenograft model using shControl and shRNPEPL1 cells. Compared with the shControl group, tumors derived from shRNPEPL1 cells were markedly smaller at the endpoint (Figure 6J). Consistently, tumor growth curves showed that RNPEPL1 knockdown significantly reduced tumor volume over time (Figure 6K). In addition, tumor weight was significantly lower in the shRNPEPL1 group than in the shControl group (Figure 6L). These findings further support the tumor-promoting role of RNPEPL1 in LUAD and strengthen the biological relevance of RNPEPL1 as a key gene in the TRPM4-related prognostic model.

### 3.7. Potential Drug Sensitivity and Experimental Validation in LUAD Cells

**Figure 7:**
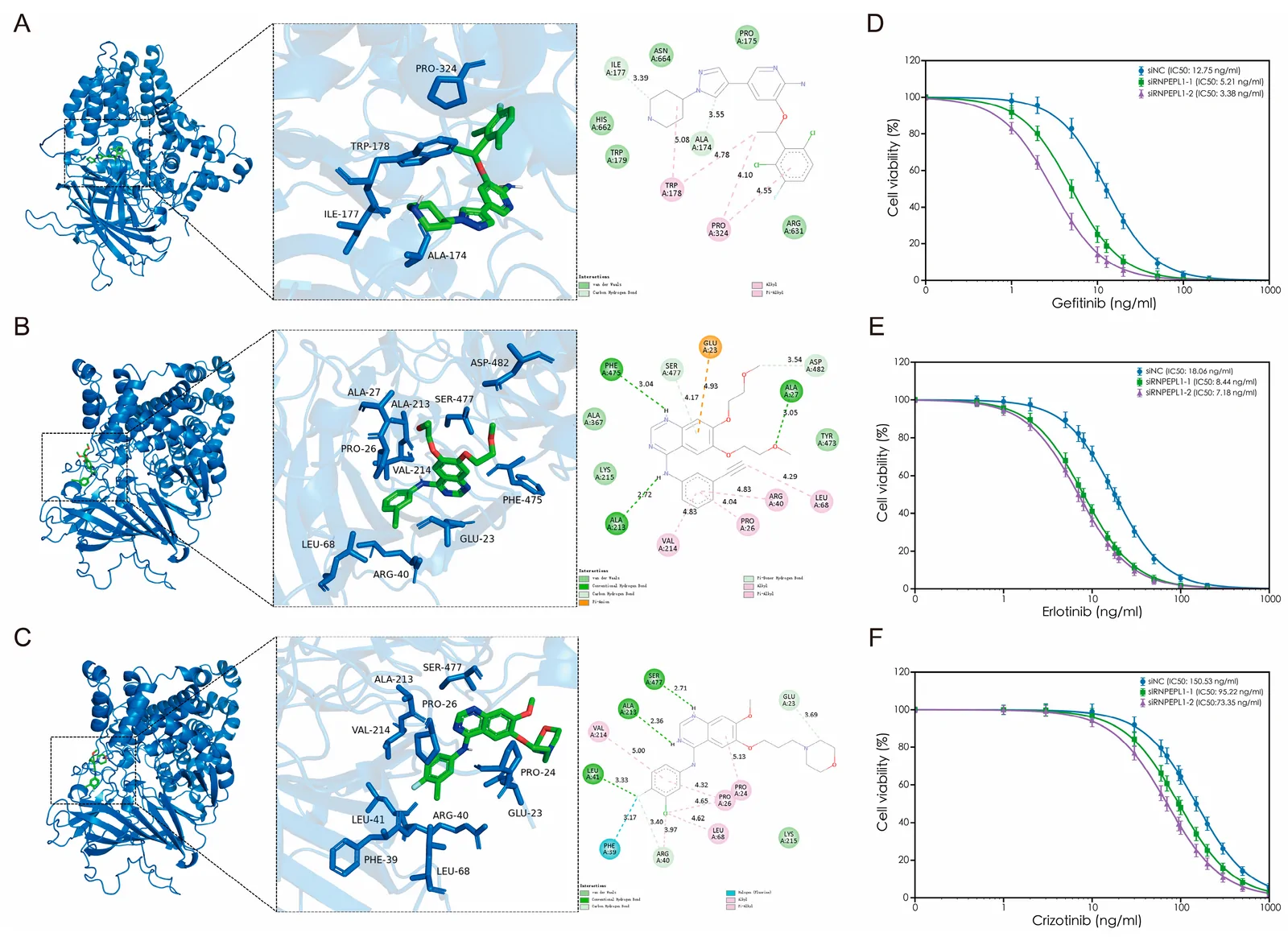
Molecular docking analysis and experimental validation of drug sensitivity in LUAD. (**A**–**C**) Molecular docking analysis showing the predicted binding patterns of gefitinib (**A**), erlotinib (**B**), and crizotinib (**C**) with RNPEPL1. The predicted binding energies were −7.8 kcal/mol for gefitinib, −7.7 kcal/mol for erlotinib, and −8.3 kcal/mol for crizotinib. Key interactions included hydrogen bonds, carbon-hydrogen bonds, hydrophobic interactions, and van der Waals contacts. (**D**) In vitro drug sensitivity assay showing the effect of RNPEPL1 knockdown on the IC50 value of gefitinib in PC9 cells. (**E**) In vitro drug sensitivity assay showing the effect of RNPEPL1 knockdown on the IC50 value of erlotinib in PC9 cells. (**F**) In vitro drug sensitivity assay showing the effect of RNPEPL1 knockdown on the IC50 value of crizotinib in H3122 cells.

To further evaluate the potential therapeutic relevance of these findings, molecular docking analysis was performed between RNPEPL1 and selected targeted agents. The docking results showed favorable binding affinities between RNPEPL1 and gefitinib, erlotinib, and crizotinib, with binding energies of −7.8, −7.7, and −8.3 kcal/mol, respectively (Figure 7A–C). Gefitinib formed interactions with residues including SER477, ALA213, LEU41, PHE39, VAL214, PRO24, PRO26, and LEU68. Erlotinib interacted with residues including PHE475, ALA213, ALA27, GLU23, ASP482, ARG40, LEU68, PRO26, and VAL214. Crizotinib interacted with residues including ILE177, ALA174, TRP178, PRO324, ASN664, HIS662, TRP179, CYS201, and ARG631. These results suggest that the three targeted agents may have potential binding activity with RNPEPL1, although these docking results should be interpreted as computational predictions requiring further biochemical validation.

Furthermore, in vitro drug sensitivity assays were conducted to provide partial experimental support for the computational predictions. After RNPEPL1 knockdown, PC9 cells showed increased sensitivity to gefitinib and erlotinib, as reflected by reduced IC50 values compared with the siNC group (Figure 7D,E). For gefitinib, the IC50 decreased from 12.75 ng/mL in the siNC group to 5.21 ng/mL and 3.38 ng/mL in the siRNPEPL1-1 and siRNPEPL1-2 groups, respectively. For erlotinib, the IC50 decreased from 18.06 ng/mL to 8.44 ng/mL and 7.18 ng/mL, respectively. In H3122 cells, RNPEPL1 knockdown also enhanced sensitivity to crizotinib, with the IC50 decreasing from 150.53 ng/mL in the siNC group to 95.22 ng/mL and 73.35 ng/mL in the siRNPEPL1-1 and siRNPEPL1-2 groups, respectively (Figure 7F). Taken together, these results suggest that RNPEPL1 may be associated with drug sensitivity-related phenotypes in LUAD cells. However, these in vitro experiments do not replace validation of the TRPM4-related risk model in clinically annotated treatment-response cohorts.

## 4. Discussion

Lung adenocarcinoma (LUAD) remains a major clinical challenge because of its marked molecular and microenvironmental heterogeneity, despite substantial advances in targeted therapy and immunotherapy for non-small cell lung cancer [1,2,3]. Recent epidemiologic and clinical reviews continue to underscore the high global burden of lung cancer and the need for biomarkers that can improve risk stratification and therapeutic selection [1,2,3]. In this context, single-cell RNA sequencing has become an important tool for resolving LUAD cellular ecosystems and for identifying biologically meaningful programs that are masked in bulk transcriptomic analyses [13,14].

In the present study, we focused on TRPM4, a key ion-channel gene increasingly implicated in sodium overload-associated cell death [15,16]. Recent literature has framed this process as necrosis by sodium overload (NECSO), a regulated necrotic program initiated by persistent TRPM4 opening, intracellular Na^+^ accumulation, reversal of Na^+^/Ca^2+^ exchange, mitochondrial dysfunction, and eventual membrane rupture [17]. At the same time, recent pan-cancer studies suggest that TRPM4 has context-dependent roles in cancer, linking it not only to sodium overload-related cell death but also to tumor immune evasion and therapeutic response [18,19,20]. Our single-cell analysis showed that TRPM4 was enriched in malignant cells and selected immune cell subsets, supporting the idea that TRPM4 should be interpreted in a cell-state-specific manner rather than solely as a bulk-level marker. This is in line with recent work showing that TRPM4 expression is associated with altered immune infiltration and that, in LUAD tissues, higher TRPM4 expression is negatively associated with T-cell and M1 macrophage infiltration. It should be noted that sodium overload-related cell death/NECSO is still an emerging concept, and the terminology has not yet been universally adopted in the regulated cell death field. Therefore, our findings should be interpreted within this evolving biological framework rather than as evidence for a fully established and universally accepted cell death category.

Based on TRPM4-associated genes, we established and externally validated a prognostic signature for LUAD. This strategy is supported by recent studies showing that integration of single-cell and bulk RNA-seq data can generate robust prognostic models and improve biological interpretability in LUAD [21]. Notably, the high-risk group identified in our study was enriched for cell cycle- and DNA replication-related pathways [22]. This finding is biologically plausible and consistent with recent LUAD studies reporting that DNA replication programs are closely linked to aggressive molecular subtypes and unfavorable prognosis. Thus, our model appears to capture not only prognostic information but also a proliferative tumor state associated with more malignant behavior.

Another important finding of our study is the potential therapeutic relevance of the TRPM4-related risk model [15,23,24]. We observed that low-risk patients showed lower computationally predicted IC_50_ values for several FDA-approved drugs used in lung cancer, including EGFR tyrosine kinase inhibitors and platinum/taxane-based agents. However, these drug-response results were inferred from transcriptomic profiles using GDSC-based cell-line pharmacogenomic models rather than from observed clinical treatment outcomes. Therefore, they should be interpreted as exploratory and hypothesis-generating rather than as direct evidence of treatment efficacy or clinical treatment selection. Although recent studies have linked TRPM4 to broad alterations in drug responsiveness, worse anti-PD-(L)1 treatment outcomes, and chemoresistance phenotypes in other tumor types, prospective validation in LUAD cohorts with detailed treatment and response information is required before the TRPM4-related signature can be considered for therapeutic decision-making [25,26,27].

Among the genes included in our model, RNPEPL1 emerged as the most important contributor in the random forest analysis. Compared with TRPM4, the role of RNPEPL1 in lung cancer remains poorly characterized in the current literature [16,28]. However, available evidence from other malignancies suggests that this gene may be clinically relevant: NPEPL1/RNPEPL1 expression has been associated with tumor growth and metastasis in colorectal cancer [29,30,31]. Our in vitro data extend these observations by showing that RNPEPL1 knockdown suppresses proliferation, colony formation, migration, and invasion in LUAD cells, supporting a tumor-promoting role in this disease. Given its recurrent appearance in aggressive tumor settings, it is reasonable to speculate that RNPEPL1 may participate in proteostasis- or matrix-remodeling-related programs, although this mechanistic link still needs to be tested directly in LUAD.

This study has several limitations. First, the prognostic model was developed and validated using retrospective publicly available datasets, which may introduce selection bias and dataset-specific heterogeneity. Second, although external validation was performed in an independent GEO cohort, prospective multicenter clinical validation is still required before clinical implementation. Third, the available public datasets lacked complete information on treatment response, progression-free survival, molecular subtypes, and immunotherapy-related biomarkers, limiting our ability to evaluate the predictive value of the signature in clinically defined treatment subgroups. In addition, the datasets used in this study did not contain complete and uniformly annotated information on established actionable molecular alterations, including EGFR, KRAS, ALK, BRAF, MET, RET, HER2, or PD-L1 status. Several clinically important alterations, such as ALK/RET fusions, MET exon 14 skipping, and PD-L1 expression, cannot be reliably inferred from the transcriptomic and survival matrices used for model construction and validation. Therefore, we could not determine whether the TRPM4-related signature provides prognostic or predictive information independent of currently established molecular classifications. Fourth, the drug sensitivity analyses were based on computational prediction using pharmacogenomic cell-line models rather than observed clinical treatment outcomes and should therefore be regarded as hypothesis-generating. The RNPEPL1 knockdown drug assays provided partial experimental support for the potential association between this key model gene and drug sensitivity, but they did not validate the full prognostic signature in clinical treatment-response cohorts. Finally, although RNPEPL1 was selected as the top-ranked model gene and experimentally validated, the remaining genes included in the prognostic signature were not functionally validated in this study. Future studies should investigate the biological roles of these genes and further clarify the mechanistic relationships among TRPM4, RNPEPL1, sodium overload-related cell death, and LUAD progression. Future studies using clinically annotated LUAD cohorts with comprehensive genomic profiling, PD-L1 assessment, treatment information, and response data are needed to clarify the relationships between this signature and actionable oncogenic drivers.

## 5. Conclusions

Our study provides a single-cell-informed and clinically relevant view of TRPM4 in LUAD. We demonstrate that TRPM4 is associated with distinct cellular distributions, adverse biological programs, and prognostic stratification, while RNPEPL1 acts as a functionally important downstream candidate with tumor-promoting effects. These findings broaden the understanding of TRPM4-related sodium overload biology in LUAD and may offer a useful framework for prognostic assessment and individualized therapeutic decision-making.

## Data Availability

The data analyzed during this study are available from the corresponding author upon reasonable request.

## Supplementary Materials

The following supporting information can be downloaded at https://www.mdpi.com/article/10.3390/cancers18162643/s1: Figure S1: Univariate and multivariate Cox regression analyses of the TRPM4-related risk score and clinicopathological factors in the TCGA cohort; Figure S2: Quantitative analysis of RNPEPL1 and TRPM4 protein expression after RNPEPL1 knockdown in LUAD cells; Figure S3: Original Western blot images; Table S1: Baseline clinicopathological characteristics of the TCGA training cohort and GSE68465 validation cohort.

## Abbreviations

LUAD, lung adenocarcinoma; TRPM4, transient receptor potential melastatin 4; RNPEPL1, arginyl aminopeptidase-like 1; TCGA, The Cancer Genome Atlas; GEO, Gene Expression Omnibus; scRNA-seq, single-cell RNA sequencing; UMAP, Uniform Manifold Approximation and Projection; PCA, principal component analysis; GO, Gene Ontology; KEGG, Kyoto Encyclopedia of Genes and Genomes; GSEA, gene set enrichment analysis; LASSO, least absolute shrinkage and selection operator; Cox, proportional hazards regression; ROC, receiver operating characteristic; AUC, area under the curve; IC50, half-maximal inhibitory concentration; CCK-8, Cell Counting Kit-8; qRT-PCR, quantitative real-time polymerase chain reaction; siRNA, small interfering RNA; OS, overall survival; SD, standard deviation; NECSO, necrosis by sodium overload.
