# Supplementary material for: Integrated Analysis Identifies a TRPM4-Related Sodium Overload Signature with Prognostic Value and Experimentally Validates RNPEPL1 in Lung Adenocarcinoma
Source: Cancers (Basel). 2026 Aug 16;18(16):2643. doi: 10.3390/cancers18162643 (PMC13511690; doi:10.3390/cancers18162643)
Supplement: Supplementary file 1 [file cancers-18-02643-s001.zip › cancers-4409854-supplementary.pdf]

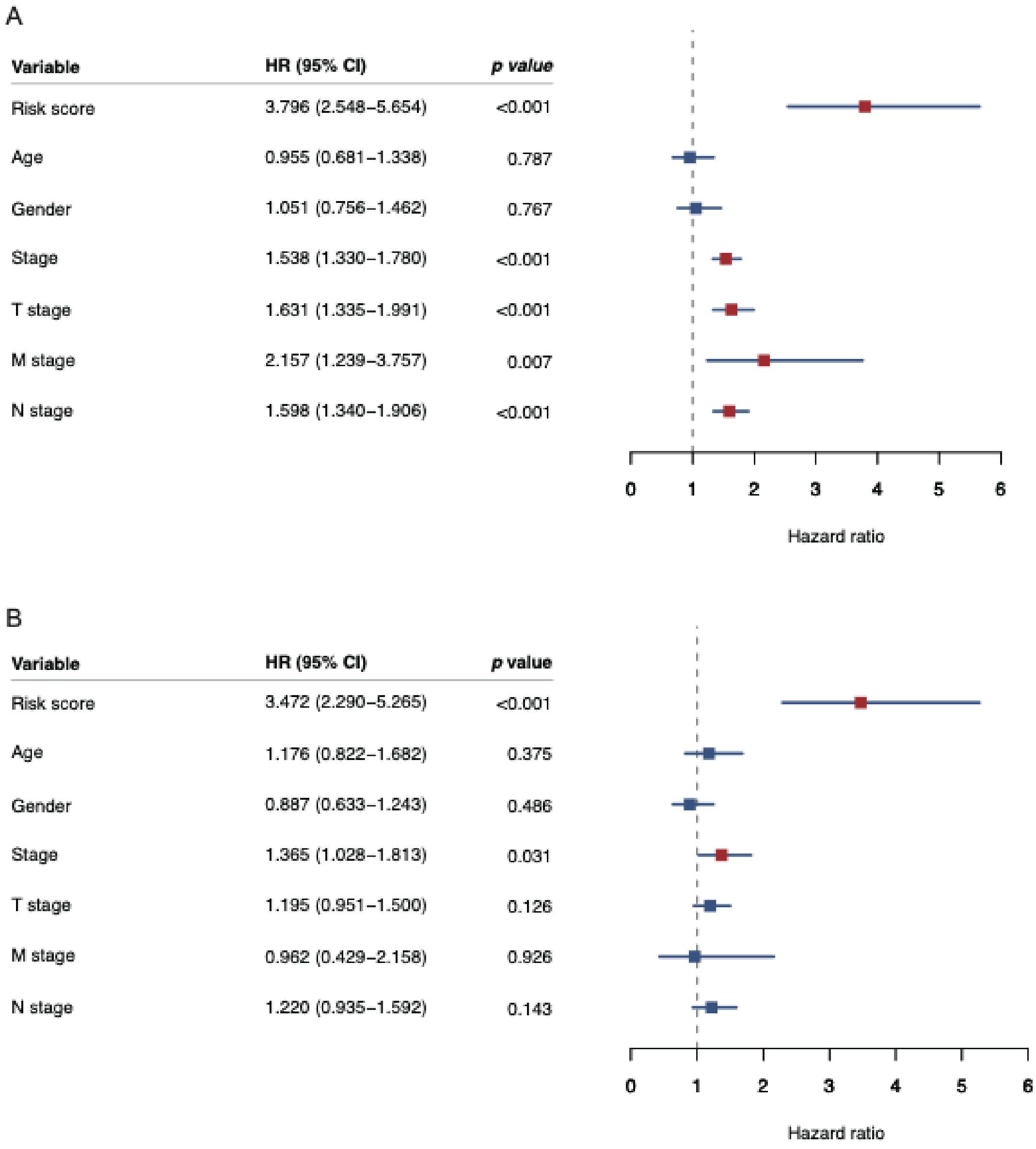

Supplementary Figure S1. Univariate and multivariate Cox regression analyses of the TRPM4-related risk score and clinicopathological factors in the TCGA cohort. (A) Univariate Cox regression analysis of the risk score, age, gender, stage, T stage, M stage, and N stage. (B) Multivariate Cox regression analysis adjusted for age, gender, stage, T stage, M stage, and N stage. HR, hazard ratio; CI, confidence interval.

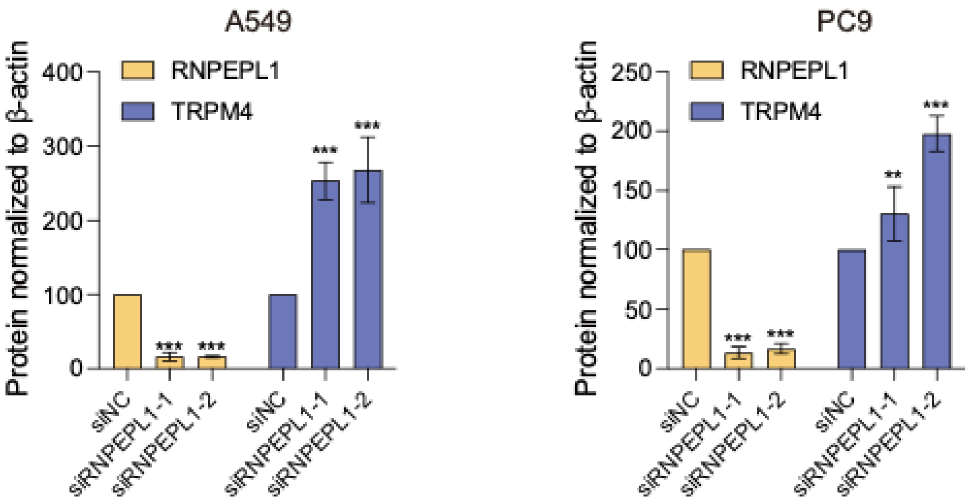

Supplementary Figure S2. Quantitative analysis of RNPEPL1 and TRPM4 protein expression after RNPEPL1 knockdown in LUAD cells. Densitometric quantification showed that siRNPEPL1 markedly reduced RNPEPL1 protein expression and increased TRPM4 protein expression in both A549 and PC9 cells. Data are presented as the mean ± SD.

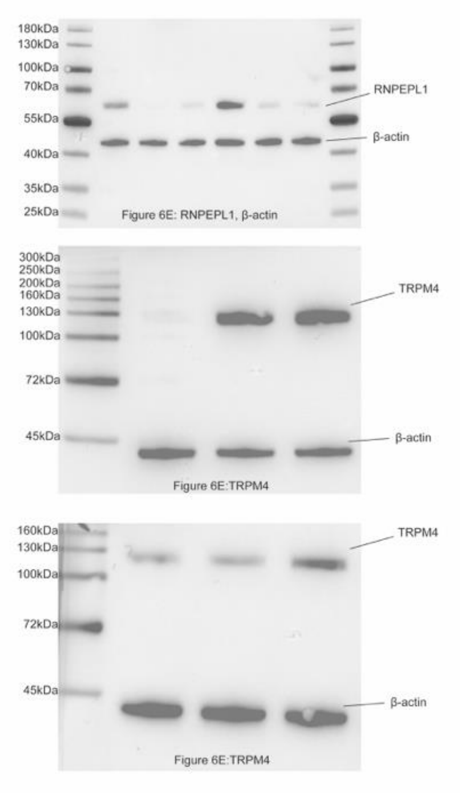

Supplementary Figure S3. Original western blot images for Figure 6E.

Supplementary Table S1. Baseline clinicopathological characteristics of the TCGA training cohort and GSE68465 validation cohort

Characteristic	TCGA_training	GSE68465_validation
Patients, n	503	442
Age, years, median (IQR)	66.0 (59.0-72.0)	65.0 (58.0-72.0)
Sex: Female	272 (54.1%)	219 (49.5%)
Sex: Male	231 (45.9%)	223 (50.5%)
AJCC stage: I	266 (54.3%)	NA
AJCC stage: II	119 (24.3%)	NA
AJCC stage: III	80 (16.3%)	NA
AJCC stage: IV	25 (5.1%)	NA
T stage: T1	168 (33.4%)	150 (34.2%)
T stage: T2	269 (53.5%)	250 (57.1%)
T stage: T3	45 (8.9%)	28 (6.4%)
T stage: T4	18 (3.6%)	10 (2.3%)
N stage: N0	326 (64.9%)	297 (67.8%)
N stage: N1	95 (18.9%)	87 (19.9%)
N stage: N2	69 (13.7%)	53 (12.1%)
N stage: N3	2 (0.4%)	0 (0.0%)
N stage: NX	10 (2.0%)	1 (0.2%)
M stage: M0	335 (67.1%)	NA
M stage: M1	24 (4.8%)	NA
Smoking: Never smoked	NA	49 (14.0%)
Smoking: Smoked in the past	NA	268 (76.8%)
Smoking: Currently smoking	NA	32 (9.2%)
Adjuvant chemotherapy: No	NA	340 (79.3%)
Adjuvant chemotherapy: Yes	NA	89 (20.7%)
Adjuvant radiotherapy: No	NA	363 (84.8%)
Adjuvant radiotherapy: Yes	NA	65 (15.2%)
Follow-up, months, median (IQR)	21.6 (13.9-37.2)	47.0 (24.0-73.7)
Vital status: Alive	321 (63.8%)	206 (46.6%)
Vital status: Dead	182 (36.2%)	236 (53.4%)
